# The performance of dipole charge density mapping integrated with robotic magnetic navigation in the treatment of atrial tachycardias

**DOI:** 10.1007/s10840-023-01552-6

**Published:** 2023-04-20

**Authors:** Rita B. Gagyi, Sing-Chien Yap, Anna M. E. Noten, Sip Wijchers, Tamas Szili-Torok

**Affiliations:** Thoraxcenter, Department of Clinical Electrophysiology, Postbus 2040, 3000 CA Rotterdam, Molewaterplein 40, 3015 GD Rotterdam, Erasmus, MC The Netherlands

**Keywords:** Atrial tachycardia, Noncontact mapping, Robotic magnetic navigation, Catheter ablation

## Abstract

**Background:**

Catheter ablation (CA) has become a well-established first-line therapy for a broad spectrum of arrhythmias, including atrial tachycardias (ATs). In this study we aimed to assess the performance of the integrated novel high-resolution new generation noncontact mapping system (AcQMap) with robotic magnetic navigation (RMN) system in CA procedures for patients with ATs including comparing patient subgroups based on the utilized mapping modality, arrhythmia mechanism, localization and type of procedure.

**Methods:**

All patients undergoing CA for AT using the AcQMap-RMN system were included. Procedural safety and efficacy were characterized by intra- and post-procedural complications. Acute procedural success and the long-term outcome were assessed in the overall group and in the subgroups.

**Results:**

A total number of 70 patients were referred for CA with atrial arrhythmias including 67 AT/AFL (mean age 57.1 ± 14.4 years), and 3 additional patients with inappropriate sinus tachycardia. Thirty-eight patients had de novo AT, 24 had post-PVI AT including 2 patients with perinodal AT, and 5 had post-MAZE AT. Two patients (2.9%) suffered post-procedural complications including 1 patient with groin hematoma and 1 patient with a transient ischemic attack. Acute success was achieved in 63/67 (94.0%) procedures. Thirteen patients (19.4%) had documented recurrence at the end of the 12-months follow-up period. The performance of AcQMap was equally good in focal vs. reentry mechanisms (p = 0.61, acute success), in the left and right atrium (p = 0.21).

**Conclusions:**

AcQMap-RMN integration might improve success rates in CA of ATs with low number of complications.

**Supplementary Information:**

The online version contains supplementary material available at 10.1007/s10840-023-01552-6.

## Introduction

Catheter ablation (CA) has become a well-established first-line therapy for a broad spectrum of arrhythmias, including atrial tachycardias (ATs) [1, 2]. Robotic magnetic navigation (RMN) guided approach allows for precise computer-guided catheter movements, and its atraumatic catheter design has a superior safety profile without compromising the efficiency of CA [3–6]. In the recent past, a novel high-resolution noncontact mapping system (AcQMap, Acutus Medical, Carlsbad, CA, USA) was introduced, which is fully integrated with the RMN system (AcQMap-RMN) allowing mapping of atrial arrhythmias with promising initial results [7, 8]. The AcQMap system uses two noncontact mapping modalities. Single position mapping (SPM) has been developed to map both regular and irregular rhythms. This approach requires only short acquisition times, while the more recently developed novel aggregated multi-position noncontact mapping algorithm (AMPM, SuperMap) targets organized atrial rhythms. AMPM aligns noncontact data from multiple catheter positions in close proximity to the cardiac chamber wall and allows for rapid diagnosis of AT mechanisms and identification of critical ablation sites [9].

In this study we set out to assess the performance of the integrated AcQMap-RMN system in CA procedures for patients with ATs, including comparison of the available mapping modalities. Further, we aimed to test the performance based on arrhythmia mechanism, localization, and type of procedure.

## Methods

### Patient inclusion and study groups

The study included patients 18 to 80 years of age with AT undergoing de novo or redo CA procedure using the integrated AcQMap-RMN system. The inclusion criteria were documented AT on 12-lead ECG, Holter monitoring, or previous CA procedure with documented recurrences. This inclusion criterion allowed to enter patients planned for de novo, post-PVI and post-MAZE procedures, too. For further subgroup analysis, the study population was divided into three groups based on the utilized mapping modality during ablation procedures as follows: group 1, SPM performed exclusively, group 2, AMPM performed exclusively, group 3, both SPM and AMPM performed. Further, patients were divided into groups based on arrhythmia mechanism, localization and type of procedure. Additionally, the study population was compared to historical procedural and outcome data of RMN guided procedures performed with sequential 3D mapping (CARTO control group). The institutional medical ethics committee approved the data collection and analysis for this study and concluded that it did not fall under the Medical Research Involving Human Subjects Act (SERCA-2, MEC-2021–0299). The authors declare that all data that support the findings of this study are available from the corresponding author upon reasonable request.

### Primary hypothesis and study design

The primary hypothesis of this study was that the AcQMap-RMN integration is safe and provides high acute and long-term success with low recurrence rates. Furthermore, we hypothesized that the novel AMPM feature offers improvements in source identification during CA procedures, leading to improved outcomes by offering high resolution in propagation maps of electrical activation. The primary endpoints of this study were safety, characterized by intra- and post-procedural complications and efficacy, characterized by acute procedural success. The secondary endpoint was procedural effectiveness characterized by recurrence during a 12-month follow-up and assessing the differences in procedure characteristics and outcome between the study groups.

### Definitions

Major complications were defined as any procedure-related adverse event, which were life threatening, requiring significant surgical intervention and prolonged hospital stay or resulted in death. Minor complications were defined as procedure-related adverse events, which resulted in minimal transient impairment of a body function or damage to a body structure, or which did not require any intervention or further therapy. We defined de novo procedures as first-time CA of AT, redo procedures as repeat procedure after one or more failed initial procedure or after documented recurrence. Total procedure time was defined as the time passed from first venous puncture until the removal of sheaths. Acute success was defined as non-inducibility of the treated arrhythmia. Recurrence was defined as any documented AT regardless of its duration. We included short-lived ATs ranging from premature atrial contractions (PACs) to 124 beats documented on 24 h-72 h Holter monitoring, when sequential mapping was unlikely possible. Short-lived ATs in our center are specifically planned with the AcQMap system.

### Data collection

Baseline demographic, clinical characteristics and procedural data from patients were collected from our prospective database using the electronic health records (HiX version 6.1) and analyzed in accordance with the hospital institutional review board policies. We collected safety data such as acute intra-procedural and post-procedural adverse events. The following demographic and procedural data were collected: age, sex, height, weight, BMI, date of procedure, procedure duration time, number of applications, application duration, radiation dose, rhythm at the end of procedure. Further, we collected and analyzed clinical data, such as left atrial dimension, left ventricular ejection fraction, comorbidities, and antiarrhythmic medication. Mapping data were collected from the AcQMap workstation.

### AcQMap and AT mapping

**Fig. 1:**
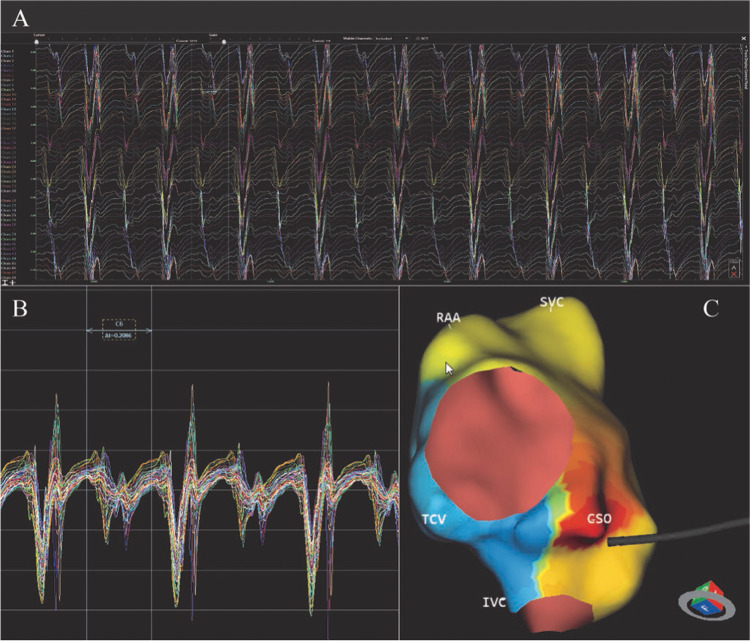
– EGMs and propagation history map (SPM). Spread EGM signals from 48 electrodes during single position mapping are shown in panel A. Stacked EGM signals are shown in panel B. Single position isochronal map is shown in panel C

**Fig. 2:**
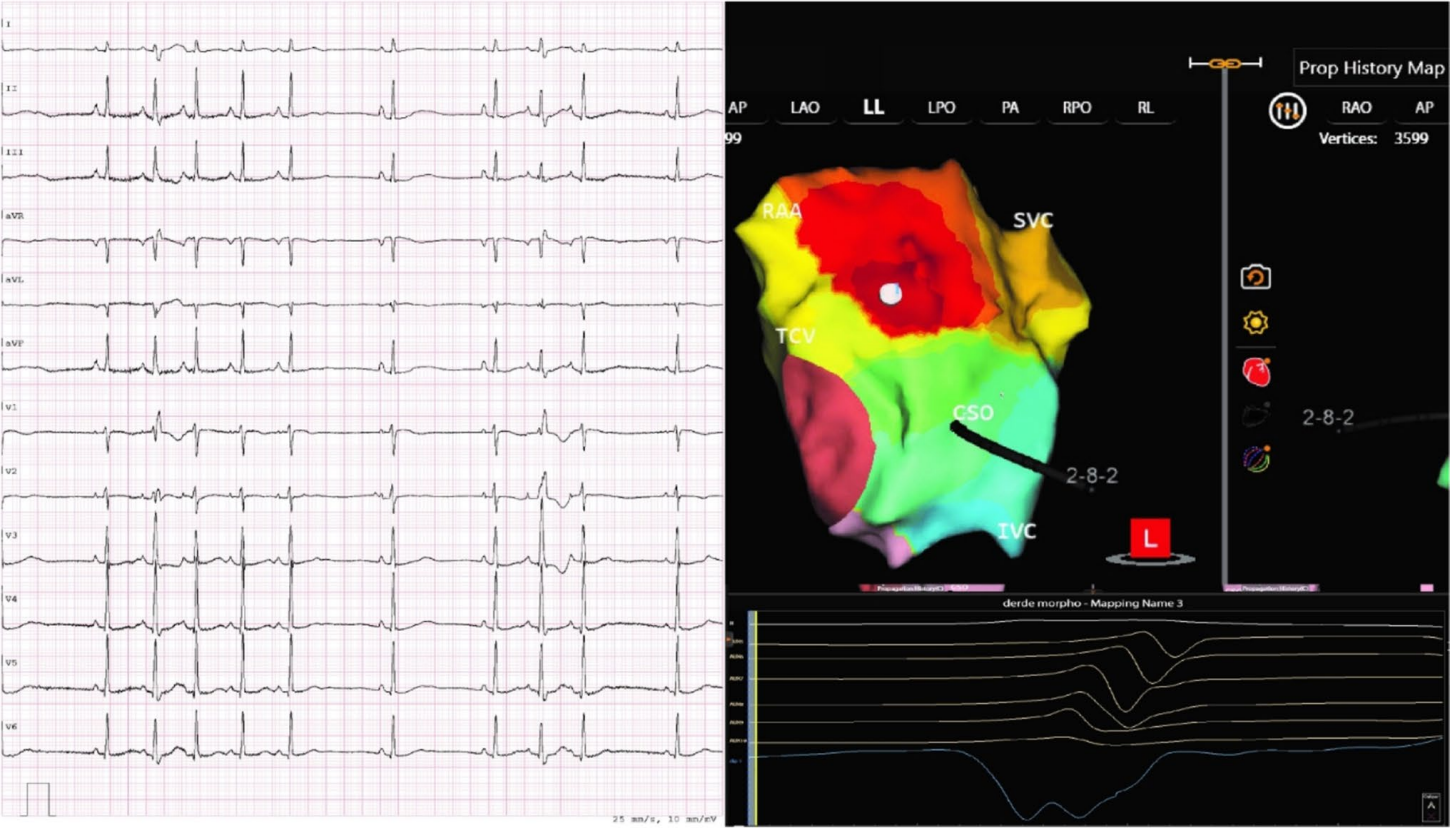
– SPM for brief episodes of AT. Patient with short-runs of AT present on ECG (on the left). Single beat single position map was acquired (right) and identified a focal firing source nearby the base of right atrial appendage (RAA)

**Fig. 3:**
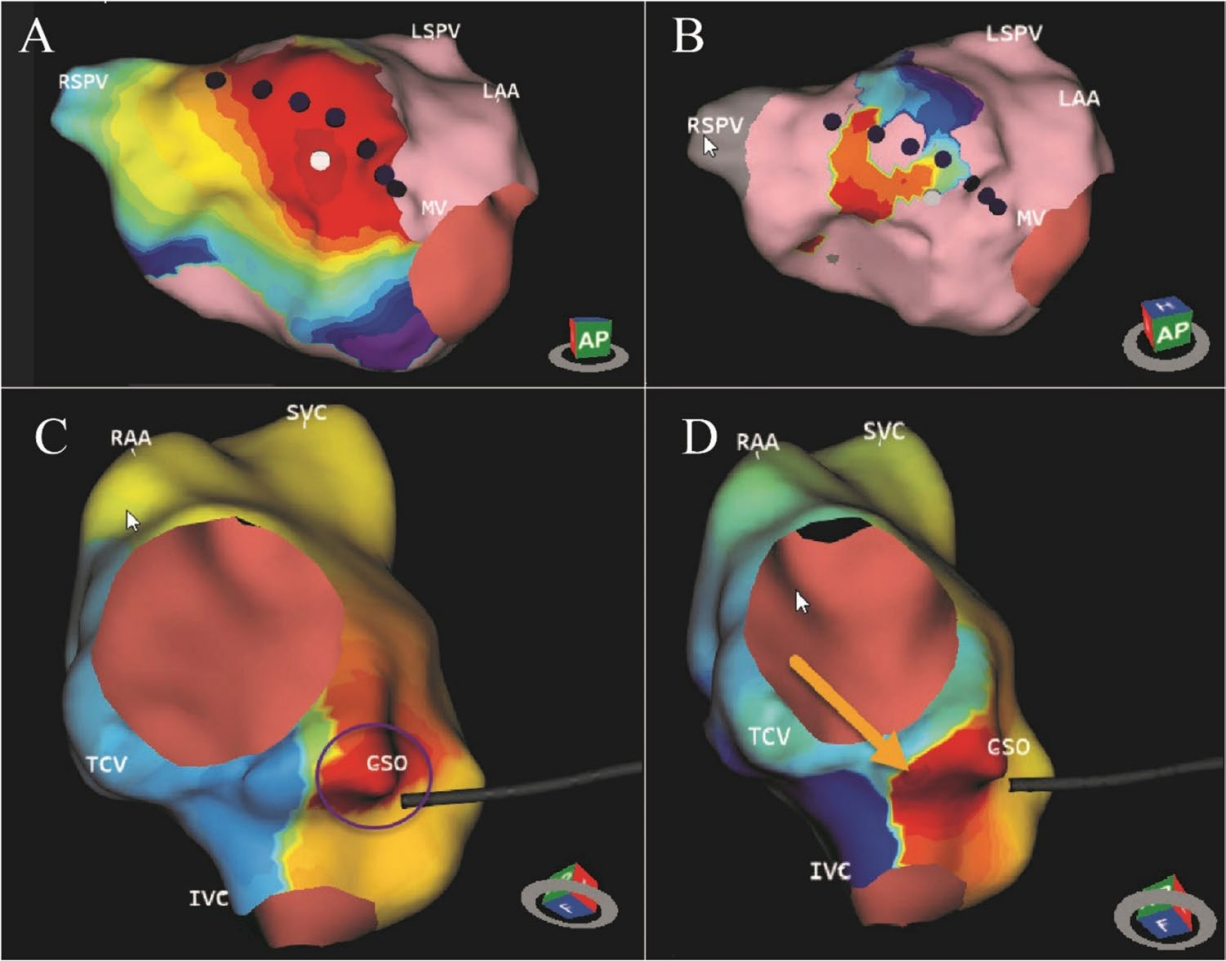
– SPM versus AMPM. Two separate cases of AT, both mapped with SPM and AMPM, too. In Panel A the SPM shows the exit of the isthmus, the propagation in CCW direction and no retrograde conduction because of the line, which contains the isthmus. However, the signal of the isthmus itself (conduction through the isthmus) is only visible on the AMPM. In panel B the SPM isochronal map shows less detail (circle), compared to the AMPM on the right, the yellow arrow shows a more detailed localization

The AcQMap system is a noncontact charge density-based mapping technology that combines ultrasound-based 3D endocardial anatomy reconstructions with high-resolution propagation history maps of electrical activation and allows visualization of global atrial activation. The 48-pole noncontact mapping catheter (AcQMap catheter, Acutus Medical, Carlsbad, CA) has six splines, each spline incorporating eight biopotential electrodes and eight ultrasound transducers. The basket catheter is manually controlled in the mapped atrium. The ultrasound-generated 3D endocardial chamber surface reconstruction corresponds to the end-diastolic size and shape of the atrium and it is created within 2–3 min. Unipolar intracardiac potentials are sensed from the biopotential electrodes of the basket catheter and are processed by an inverse solution to derive the dipolar charge sources at the endocardial surface. The waves of activation are displayed across the 3D anatomy reconstruction through time as high-resolution propagation history maps. The noncontact modality of the AcQMap system allows for two modalities of mapping: single position mapping (SPM) and aggregated multiposition noncontact mapping (AMPM). SPM can be applied as single-beat analysis to map short non-sustained ATs or PACs (Fig. 1 and 2). While AMPM enables mapping of both non-sustained and sustained repetitive atrial rhythms (**Video** 1). After hovering the basket catheter around the cardiac chamber, multiple noncontact catheter positions are time aligned based on coronary sinus (CS) activation (Fig. 3). The utilized mapping modality was chosen at the discretion of the operator if the arrhythmia lasted long enough. In cases of short-lived ATs and PACs only SPM was possible.

### Catheter ablation and follow-up

All procedures were performed using the Niobe ES robotic system (Stereotaxis, St. Louis, MO, USA). An initial standard diagnostic EP study was performed for every patient. After the presence of slow-pathway or accessory pathway mediated reentrant arrhythmias was excluded, attempts of AT induction were made including atrial programmed extrastimulation and burst atrial pacing. If this was unsuccessful or when AT did not occur spontaneously, isoproterenol was administered. The AcQMap mapping catheter was introduced via a 12.4F deflectable sheath (AcQGuide sheath, Acutus Medical, Carlsbad, CA). After reconstructing the endocardial anatomical surface, we overlaid high resolution charge density based maps of electrical activation using the AcQMap mapping system (**Video** 2). After identification of the chamber of origin, mechanism and the exact location of the substrate, ablation was performed using the MagnoFlush (Medfact, Germany) ablation catheter. The following settings were used: 45-50W (posterior wall—anterior wall, respectively), 17 mL/min flow rate, maximum 43℃. Intravenous heparin was administered for anticoagulation, guided by activated clotting time (> 350 s for LA and > 300 s for RA). We interpreted propagation history maps, identified atrial activation patterns, and performed targeted ablation. A standard of 30 min waiting time was applied to all procedures. After the procedures, patients were continuously monitored, and pre-discharge echocardiography was performed in all patients in order to exclude post-procedural complications.

A follow-up visit at 3 months after the procedure was planned for every patient. In cases where there was no recurrence at the follow-up visit, the patients were discharged and were re-evaluated 12 months after the ablation. The post-PVI/post-MAZE AT patients were followed up based on the PVI follow-up protocol with a routine 6- and 12-month hospital visit. During the follow-up visits 24-h (3 and 6 months) and 7-day Holter recordings (12 months) were analyzed for documentation of recurrent arrhythmias. Patients with recurrences were considered for repeated CA procedure or were identified as ablation failures.

### Statistical analysis

Categorical variables were described as percentages, whereas continuous variables were expressed as mean ± SD for normally distributed data or as medians (interquartile range /IQR) for skewed data. Groups were compared using the student t test, the Mann–Whitney U test, and ANOVA analysis, respectively. Statistical analysis was performed using the SPSS (version 25.0) software.

## Results

### Demographics and patient data

A total of 70 consecutive patients were included using the AcQMap-RMN system including 67 AT (mean age 57.1 ± 14.4 years), and 3 additional patients with inappropriate sinus tachycardia (IAST). IAST patients were excluded from the analysis. Out of 67 patients, 38 had de novo AT (56.7%). From the remaining patients, 24 had post-PVI AT (35.8%) including 8 patients with perinodal origin, and 5 had post-MAZE AT (7.5%). In total, 39 patients were mapped with single position mapping only (group 1, n = 39), 12 patients with AMPM exclusively (group 2, n = 12), and 16 patients with both single position and AMPM (group 3, n = 16). There were no differences in demographics and pre-operative clinical and echocardiography data between the patient groups (Table 1). Twenty-eight patients had short-lived ATs (duration ranging from a single PAC to 124 beats) and were mapped exclusively by SPM. The average reported cycle length of short-lived ATs was 370 ± 90 ms and median mapping time during the procedures was 3 min 40 s (interquartile range 2 min 15 s – 4 min 29 s).

**Table 1:** Demographics and patient characteristics

	Overall N = 67	Group 1 N = 39	Group 2 N = 12	Group 3 N = 16	*P*-value
Age (years)	57.7 ± 14.2	54.7 ± 15.3	66.8 ± 9.6	58.8 ± 11.3	0.72
Male (female)	39 (28)	22 (17)	10 (2)	7 (9)	0.48
BMI	26.9 ± 3.9	26.6 ± 4.2	28.6 ± 4.0	26.5 ± 3.3	0.49
Previous PVI	24 (33.8)	10 (25.6)	5 (45.5)	7 (50.0)	0.29
Previous MAZE ablation	5 (7.7)	2 (5.1)	2 (18.2)	1 (7.1)	0.38
Hypertension (%)	22 (34.4)	13 (34.2)	4 (36.4)	5 (35.7)	0.92
Heart failure (%)	3 (4.7)	1 (2.6)	1 (9.1)	1 (7.1)	0.35
Ischemic heart disease (%)	7 (10.9)	6 (15.8)	0 (0.0)	1 (7.1)	0.09
Cardiomyopathy (%)	2 (3.1)	2 (5.3)	0 (0.0)	0 (0.0)	0.71
Diabetes (%)	8 (12.5)	2 (5.3)	3 (27.3)	3 (21.4)	0.11
Dyslipidemia (%)	2 (3.1)	2 (5.3)	0 (0.0)	0 (0.0)	0.48
CVA/TIA (%)	10 (15.6)	5 (13.2)	1 (9.1)	4 (28.6)	0.44
OSAS (%)	4 (6.3)	2 (5.3)	1 (9.1)	1 (7.1)	0.64
AAD (%)	29 (45.2)	14 (36.8)	8 (72.7)	7 (50.0)	0.10
LVEF (%)	54.2 ± 6.4	54.4 ± 7.5	55.7 ± 4.1	52.1 ± 4.8	0.51
LA diameter (mm)	44.9 ± 13.6	41.8 ± 7.5	48.2 ± 5.4	48.9 ± 22.3	0.41
LAVI	39.6 ± 12.0	39.5 ± 14.1	39.4 ± 8.6	40.5 ± 11.1	0.49
TAPSE	19.6 ± 5.8	18.9 ± 6.7	19.9 ± 5.2	20.7 ± 4.4	0.40

Values are given as mean ± SD, n (%). BMI indicates body mass index; AAD, antiarrhythmic drug; LA, left atrium; LVEF, left ventricular ejection fraction; LAVI, left atrial volume index; IHD, Ischemic heart disease; CVA/TIA, cerebrovascular accident/transient ischemic attack; OSAS, obstructive sleep apnea syndrome; TAPSE, tricuspid annular plane systolic excursion

For additional comparison, the control group consisted of 58 patients with AT mapped and ablated using sequential 3D mapping integrated with RMN (CARTO, Biosense Webster, Irvine, CA, USA) (mean age 56.3 ± 15.0 years). In the CARTO control group 39 patients had de novo AT (67.2%), 11 had post-PVI AT (19.0%) and 8 patients had post-MAZE AT (13.8%).

### Procedural data summary

In the overall patient group, the average procedure duration was 174.7 ± 61.8 min (mean ± SD). There were no differences in procedure duration between group 1, group 2 and group 3 (165.9 ± 55.9, 168.7 ± 75.6, and 188.5 ± 47.9, P = 0.70; values are mean ± SD). Fluoroscopy dose in group 1 was less than in group 2 (133.0 vs. 166.3 mGy, p = 0.01). The overall median radiofrequency application number was 24.0 (IQR 10.7—48.3). Arrhythmia termination was achieved in 61/67 patients (88.4%), electrical cardioversion (ECV) was performed in 4 patients (5.9%). Detailed report on procedural data is presented in Table 2. Comparison with historical control group is detailed in Table 3.

**Table 2:** Procedural data

	Overall	Group 1	Group 2	Group 3	*P*-value
Fluoroscopy dose* (mGy)	152.0 (84.3 – 299.5)	133.0 (72.0–188.5)	166.3 (119.0–324.0)	162.0 (108.2–411.0)	0.80
Procedure duration (min)	174.7 ± 61.8	171.2 ± 59.3	170.4 ± 82.5	190.0 ± 53.3	0.70
Application number* (n)	24.0 (10.8 – 48.3)	22.0 (9.0 – 39.0)	26.0 (16.0 – 61.0)	38.0 (19.8 – 62.3)	0.06
Application duration* (s)	964.0 (425.5 – 1724.5)	717.5 (325.8 – 1593.0)	1573.9 (698.0 – 3296.0)	1476.5 (785.0 – 1946.3)	0.90
Waiting time (min)	16.8 ± 10.8	16.5 ± 11.0	18.2 ± 10.8	16.4 ± 10.6	0.77

Values are given as mean ± SD, n (%), and * values are presented as median and interquartile range (IQR)

**Table 3:** Comparison with control group

	AcQMap *n* = 67	Control *n* = 58	*P*-value
Fluoroscopy dose* (mGy)	152.0 (84.3 – 299.5)	132.5 (31.3–270.5)	0.10
Procedure duration (min)	174.7 ± 61.8	147.5 ± 51.1	0.01
Application number* (n)	24.0 (10.8 – 48.3)	23.0 (9.5 – 39.0)	0.46
Application duration* (s)	964.0 (425.5 – 1724.5)	1059.0 (357.5 – 1978.0)	0.90
Acute AT termination after RF application	61 (88.4%)	46 (79.3%)	0.04
ECV	4 (5.9%)	9 (15.5%)	0.14
Procedural complication	2 (2.9%)	5 (8.6%)	0.24
Cumulative AT recurrence	13 (19.4%)	27 (46.6%)	< 0.01

### Primary endpoint: safety and efficacy

Two patients (2.9%) had post-procedural complications including 1 patient with groin hematoma and 1 patient with transient ischemic attack. Acute success was achieved in 63/67 (94.0%) procedures. In one patient ablation was not performed due to the parahisian localization of the arrhythmia source.

### Performance by AT mechanism and localization

Focal mechanism was identified in 43 patients, re-entry mechanism was identified in 21 patients and perinodal mechanism was identified in 3 patients. Acute procedural success was achieved in 41/43 (95.3%) patients with focal AT, 19/21 (90.5%) in patients with re-entry mechanism, and 3/3 (100.0%) in patients with perinodal AT (p = 0.61). There were no differences in procedural times (focal 179.1 ± 61.2, re-entry 163.8 ± 60.4, perinodal 147.7 ± 31.5 min, p = 0.48), nor in fluoroscopy doses (focal 140.0 IQR 71.0–297.0, re-entry166.5 IQR 102.5–321.0, perinodal 208.0 IQR 18.0–1116.0 mGy, p = 0.57). Out of 43 patients with focal ATs, 9 had recurrences (20.9%), out of 21 patients with re-entry ATs, 4 had documented recurrences (19.0%), and none of the patients with perinodal mechanism (0.0%) had documented recurrences during the follow-up period (p = 0.67).

**Fig. 4:**
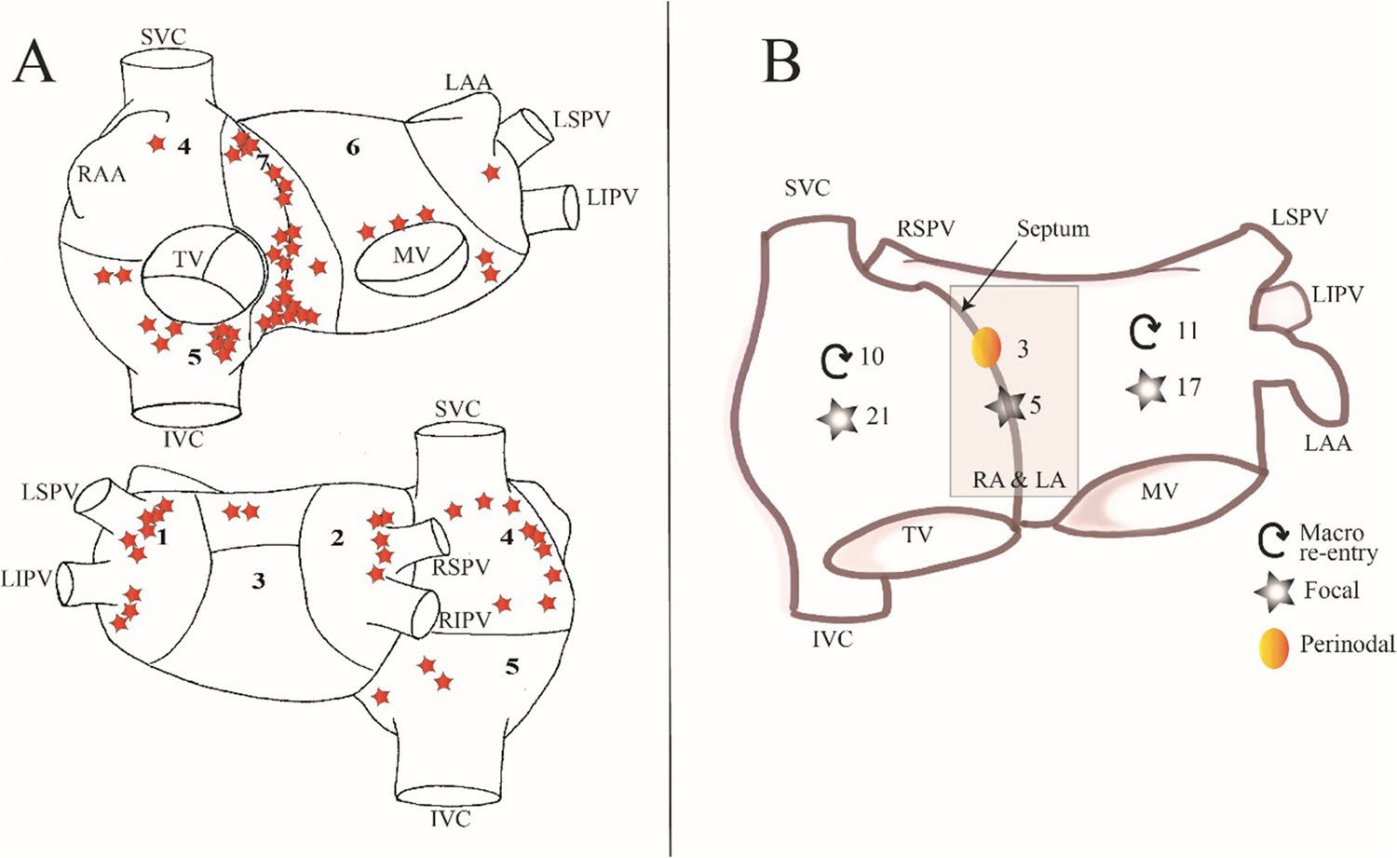
– Ablation targets. Panel A-Exact localization of the identified targets are presented on a schematic atrial illustration as red stars anterior and posterior view. Biatrial schema is divided into 7 regions. Region 1 indicating the left pulmonary veins and left appendage; region 2, right pulmonary veins and posterior interatrial groove; region 3, inferior and posterior left atrium; region 4, upper half of right atrium and RAA; region 5, lower half of right atrium; region 6, anterior left atrium and roof; and region 7, anterior interatrial groove. Panel B-Mechanism and localization of AT sources. SVC = superior vena cava, IVC = inferior vena cava, TV = tricuspid valve, RSPV = right superior pulmonary vein, LSPV = left superior pulmonary vein, LIPV = left inferior pulmonary vein, LAA = left atrial appendage, MV = mitral valve, RA = right atrium, LA = left atrium

In 31 cases the AT was localized in the RA, in 28 cases in the LA, and in 8 patients targets were localized in both RA and LA localization. Acute procedural success was achieved in 28/31 (90.3%) patients with RA, 27/28 (96.4%) patients with LA and 8/8 (100.0%) patients with RA and LA AT localization (p = 0.21). There were no differences in procedural times (RA 165.1 ± 59.8, LA 173.5 ± 53.6, RA and LA 199.4 ± 69.5 min, p = 0.42), nor in fluoroscopy doses (RA 152.0 IQR 72.5–302.3, LA 150.0 IQR 86.5–301.5, RA and LA 228.5 IQR 100.8–437.3 mGy, p = 0.72). Out of 31 patients with AT localized in the RA, 6 had documented recurrences (19.4%), 5 patients from 28 patients (17.9%) with AT localized in LA, and 2 patients out of 8 (25.0%) with AT localized in both RA and LA had documented recurrences during the follow-up period (p = 0.90). In one patient AMPM showed a difference in target localization. Mechanism and localization of ATs is illustrated on Fig. 4.

### Follow-up summary

In the overall patient group 13 patients (19.4%) had documented recurrence at the end of the 12-month follow-up period. From de novo AT/AFL patients, 6 had documented recurrence (15.8%), from the post-PVI and post-MAZE AT/AFL patients 7 had recurrence (24.1%). During the 3-months follow-up 8 patients had documented recurrence (de novo n = 1, post-PVI n = 5, post-MAZE n = 2, p < 0.01). At 6-months follow-up visits, 8 patients had documented recurrence (de novo n = 3, post-PVI n = 4, post-MAZE n = 1, p = 0.45). During the 12-months follow-up visit, 7 patients had documented recurrence (de novo n = 4, post-PVI n = 3, post-MAZE n = 0, p = 0.55). One patient underwent redo ablation with remapping utilizing the AcQMap system. During the redo procedure, the AT showed similar localization as during the initial procedure (inferolateral side of the tricuspid valve – lateral/posterior right atrial wall).

## Discussion

The main finding of this study is that the AcQMap technology may offer a safe and effective treatment option for patients with all types of ATs. The AcQMap-RMN integration might provide equal performance in different AT mechanisms, localizations and type of procedures. Our data suggest that the addition of the novel AMPM modality had only confirmatory role, and did not extend the procedure time significantly.

### Major advantages of robotic guided CA

The most essential requirements for a successful CA procedure are accurate substrate localization and optimal radiofrequency energy delivery provided by good catheter-tissue contact. Manual catheter manipulation is often challenging due to the catheter’s predefined curve, which requires additional manual skills to maneuver in some cases. The RMN technology has proven to be effective and safe in CA procedures [10, 11]. It provides precise and stable catheter manipulation, and practically eliminates acute intra-procedural complications [6, 12]. As previous studies proved, the RMN system shows superiority to conventional manual methods in ventricular tachycardia ablation [13–16]. Prior studies have shown also that using the RMN system in AT ablation procedures reduces fluoroscopy use compared to manual methods, however acute and long-term success rates demonstrated no significant improvements with the RMN system [17, 18]. The localization of the substrates in this study (Fig. 4) demonstrated wide variety of targets, some of them being localized in areas being difficult to reach with manually steered catheters. With RMN guided catheters, however, the operator is able to easily reach difficult targets such as an anterior line between the right superior pulmonary vein and mitral valve.

### Catheter ablation of atrial tachycardias

Some fundamental data are available from the period before the introduction of three-dimensional electroanatomic mapping (EAM) systems [19]. On the other hand, the introduction of EAM has revolutionized the approach to CA treatment of AT [20, 21]. It opened the perspective to truly investigate and visualize the underlying electrophysiological mechanisms and the identification of the chamber of origin [22].

Despite this, until recently sequential EAM was time consuming and therefore required stable tachycardias. Short lasting ATs or brief episodes and varying CLs were the most challenging issues. On top of that, mechanical induction of PAC and the inability of re-induction potentially threatens the adequate diagnosis. Frequent degeneration into atrial fibrillation requires multiple electrical cardioversions with its obvious disadvantages during the mapping procedure. Although high-density multielectrode mapping [23] addresses many of these concerns, it could not solved them completely. On top of that, the presence of multiple ATs per procedure requires multiple maps. Even with the fastest technology it can be time consuming and may have significant fatigue effect on the EP operator. These can clearly explain why diverse success rates are reported in the literature. Typically more standardized results are expected and reported in patients without structural heart diseases and focal origin, while with the extended indication to very complex ACHP (adult congenital heart disease) patient or post-MAZE ATs, a broader and somewhat lower rate of successes are reported. Nevertheless, the technology we report in this paper has the potential to solve most of these difficulties. Especially the availability of very high density single beat map makes the result independent from the duration and therefore also from the stability of the ongoing AT. The way in which AcQMap detects conduction velocities in the human atrium allows the operator to identify slow conductions zone very rapidly in the examined chamber. Our previous data suggested that short-lived highly symptomatic ATs can be mapped using SPM (AcQMap) and ablated successfully with high acute and long-term success rate [24]. Additionally, we found that ATs could be eliminated in a shorter period of time, when patients are directly scheduled for AcQMap-guided ablation.

### AcQMap-RMN integration in AT ablation

By integrating the AcQMap system with the RMN system we can now combine the benefits of real-time global chamber anatomical and activation mapping with the safety and accuracy of RMN. This study confirms that integrating this novel dipole charge density based mapping technology with the RMN system does not have a negative impact on the excellent safety profile. Moreover, AcQMap-RMN integration offers the possibility to choose between mapping modalities after the operator’s preferences and the characteristics of the arrhythmia. By offering rapid switches between mapping modalities, this system increases diversity and versatility. From the future robotics perspective would be important to integrate more available mapping systems that can be implemented on a very flexible way during the treatment of arrhythmia patients.

We previously showed additional benefits of the AcQMap mapping system integrated with RMN. Our data suggested that the AcQMap-RMN integration has no negative impact on the excellent safety profile of RMN guided ablations [25]. We found that AcQMap-RMN integration might be superior to the CARTO mapping system integrated with RMN in persistent atrial fibrillation and AT ablation. However, it requires longer procedure times and uses more fluoroscopy during the early learning phase [25]. Similarly to previous results, in the current study we found that our patients had less AT recurrences when mapped and ablated using the AcQMap mapping system, however procedures were more time-consuming.

### Limitations

The retrospective nature is the major limitation of the present study, although we included a relatively high number of patients with AT undergoing CA using the novel AcQMap mapping systemintegrated with RMN. The AcQMap technology requires fluoroscopic confirmation of the position of the mapping catheter, therefore radiation doses are relatively high. When compared to the control group, we found that AcQMap-RMN procedures are longer. A possible reason for this is that the AcQMap technology is at its early stage, it can face technical issues during the procedures and it has fewer automatic features. Previous data shows progressive reduction in prodeure duration and fluoroscopy use observed after a rather slow learning curve [26].

## Conclusions

Catheter ablation procedures guided by the AcQMap-RMN demonstrate excellent safety, high acute and long-term success rates, with low recurrence rates in patients with AT. With the combination of these two systems, patients may benefit from a more individualized treatment option with promising long-term results.

### Supplementary Information

Below is the link to the electronic supplementary material.

Supplementary file1 (MP4 611 KB)

Supplementary file2 (MOV 19025 KB)

## Data Availability

The data underlying this article will be shared on reasonable request to the corresponding author.
